# Optical coherence tomography and fundus autofluorescence findings in presumed congenital simple retinal pigment epithelium hamartoma

**DOI:** 10.3205/oc000078

**Published:** 2017-10-25

**Authors:** Prabu Baskaran, Dhananjay Shukla, Parag Shah

**Affiliations:** 1 Aravind Eye Hospital and Postgraduate Institute of Ophthalmology, Pondicherry, India; 2 Ratan Jyoti Netralaya, Gwalior, India; 3 Aravind Eye Hospital and Postgraduate Institute of Ophthalmology, Coimbatore, India

**Keywords:** congenital simple RPE hamartoma, RPE tumors, autofluorescence, optical coherence tomography

## Abstract

**Aim:** Presumed congenital simple retinal pigment epithelium hamartoma is a rare benign lesion of the macula that mimics congenital hypertrophy of the retinal pigment epithelium (RPE) and combined hamartoma of the retina and the RPE; newer imaging modalities can help in diagnosis. We report three patients with presumed congenital simple RPE hamartoma, and describe the enhanced-depth imaging optical coherence tomography (EDI-OCT) and fundus autofluorescence (FAF) findings.

**Methods:** Two patients were asymptomatic; one had an intraocular foreign body in addition to the hamartoma. All had a similar jet black, elevated lesion in the macula, sparing the fovea. EDI-OCT showed a characteristic hyperreflective layer with complete optical shadowing of the deeper layers; FAF showed pronounced hypoautofluorescence of the lesion.

**Conclusion:** Multimodal imaging with FAF and EDI-OCT can help to differentiate simple RPE hamartoma from similar RPE lesions, and may serve as a useful adjunct to clinical diagnosis of this rare tumor. We present the second largest series of presumed congenital simple RPE hamartoma, and – to the best of our knowledge – the first report of FAF findings of this tumor.

## Introduction

Tumors of the retinal pigment epithelium (RPE) are rare. They are classified into four groups, congenital hamartoma of the RPE, congenital hypertrophy of the RPE (CHRPE), combined hamartoma of retina and RPE (CHRRPE) and adenoma or adenocarcinoma of the RPE [1]. The differentiation between these conditions is primarily based on clinical examination. Spectral domain optical coherence tomography with enhanced depth imaging (EDI-OCT) has been described as an effective and non-invasive tool in diagnosing a variety of these RPE lesions [2], [3]. Fundus autofluorescence imaging (FAF) is a promising but seldom utilized tool for diagnosis of RPE tumors [4]. We report FAF and EDI-OCT findings in three patients with presumed congenital hamartoma of the RPE.

## Case descriptions

### Case 1

**Figure 1:**
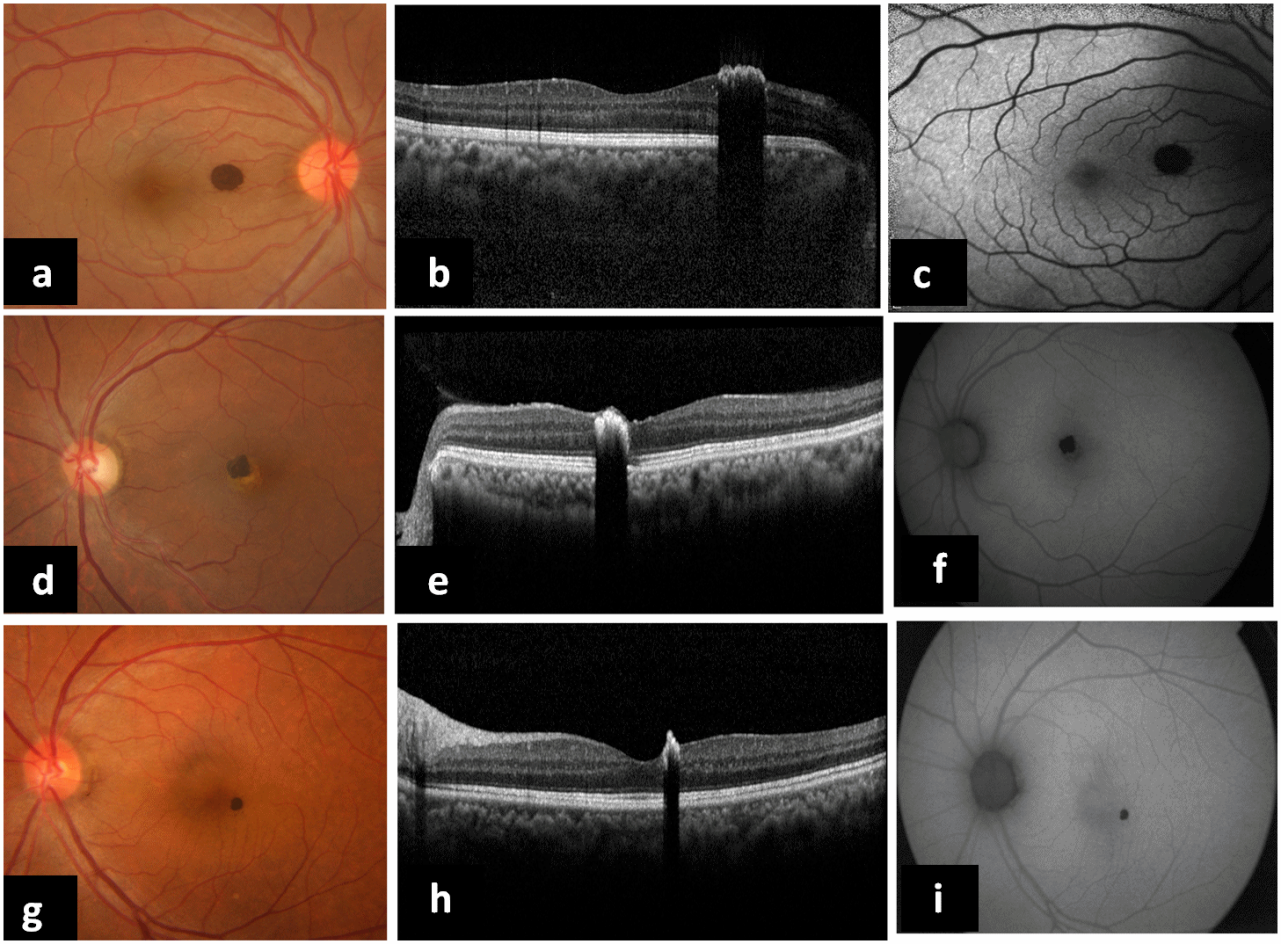
(a, d, g) Fundus photographs of case 1, case 2 and case 3 respectively, showing presumed congenital simple RPE hamartoma close to fovea without any hemorrhage or exudation in the surrounding retina. (b, e, h) EDI-OCT images showing hyperreflective surface of the lesion with total abrupt optical shadowing of the deeper layers. Choroidal thickness is normal. (c, f, i) FAF imaging showing marked, uniform hypoautofluorescence of the lesions with isoautofluorescent perimeter.

**Figure 2:**
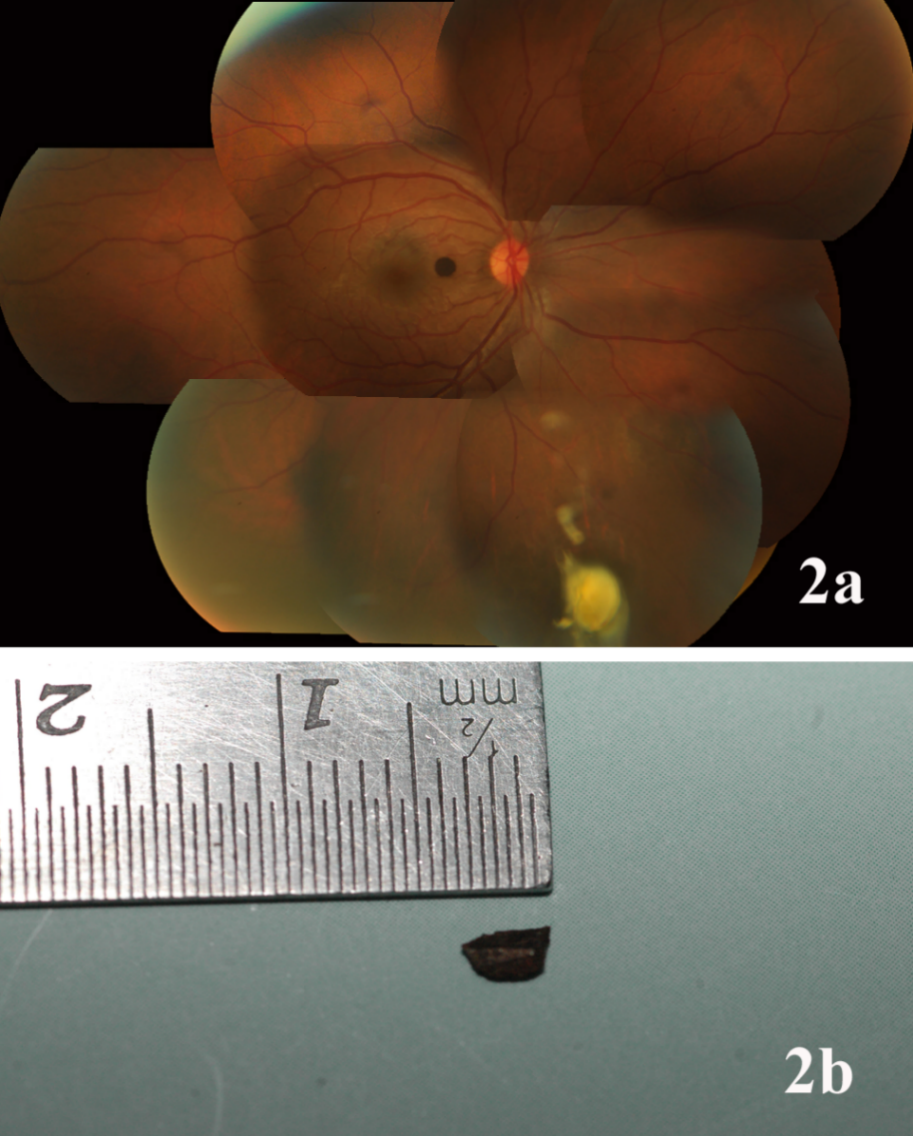
(a) Fundus montage of case 1 showing the IOFB obscured by surrounding exudates in the inferior periphery. (b) Metallic IOFB following removal by pars plana vitrectomy.

A 25-year-old man presented with pain and redness OD one week following chisel-and-hammer injury. His best-corrected visual acuity (BCVA) was 20/30 OD and 20/20 OS. Intraocular pressures (IOP) were within normal limits OU. Anterior segment examination OD showed a full-thickness self-sealed corneal tear with an underlying iris hole and focal cataract. Fundus examination revealed a single, round, elevated, jet black lesion nasal to the fovea (mimicking a foreign body) on the surface of the retina (Figure 1a (Fig. 1)). In addition, there was an intraocular foreign body (IOFB) in the vitreous cavity (inferonasal quadrant) obscured by surrounding exudates (Figure 2a (Fig. 2)). B scan ultrasonography and X-ray confirmed the presence of this IOFB and ruled out a second IOFB. Examination of OS was unremarkable. EDI-OCT was performed (Spectralis HRA + OCT; Heidelberg Engineering, Heidelberg, Germany): The macula was scanned with 19 parallel horizontal 9-mm raster lines within a 5x20 degree rectangle centered on the fovea, each line being composed of 100 averaged scans. The parafoveal lesion was evaluated for thickness, configuration, and effects on retinal and choroidal tissue. Choroidal thickness by EDI-OCT was measured manually at four sites, just adjacent to the tumor edge in the superior, nasal, inferior and temporal directions on raster lines passing through the lesion; these readings were averaged to obtain the mean choroidal thickness around the hamartoma. EDI-OCT revealed an irregular, elevated, hyperreflective lesion with complete optical shadowing of the underlying retina as well as choroid; the mean choroidal thickness was within normal limits (304 µm) around the hamartoma [5] (Figure 1b (Fig. 1)). Fundus autofluorescence was obtained using a confocal scanning laser ophthalmoscope (Heidelberg Retinal Angiograph 2, Heidelberg Engineering, Heidelberg, Germany), with an excitation wavelength of 488 nm and a barrier filter of 500 nm. A series of 50–90 images was averaged to obtain a high-quality picture. FAF imaging showed pronounced and discrete hypoautofluorescence of the lesion, with normofluorescent surrounding retina (Figure 1c (Fig. 1)). Pars plana vitrectomy with foreign body removal was performed OD (Figure 2b (Fig. 2)). On his last follow-up visit at three months, his BCVA was maintained at 20/30 with attached retina. The fundi of the parents and other siblings were examined and found to be unremarkable. 

### Cases 2 and 3

Case 2 was a 60-year-old woman who reported with poor vision OU despite undergoing cataract surgery OS. BCVA was 20/200 OD and 20/40 OS. Case 3 was a 63-year-old woman complaining of defective vision OD for six months: BCVA was 20/30 OD and 20/20 OS. Both patients had remarkably similar findings on biomicroscopy OU: A visually significant cataract OD and pseudophakia OS. Fundus examination OD was normal in both patients. Fundus examination OS revealed a jet black hamartoma similar to case 1 in both patients: close to the fovea nasally in case 2 and temporal to it in case 3 (Figure 1d,g (Fig. 1)). EDI-OCT and FAF (TRC-50EX mydriatic camera, Topcon medical systems, Tokyo, Japan) findings in these cases were similar to the case 1 (Figure 1e–f,h–i (Fig. 1)). The choroidal thickness around the hamartoma was unremarkable in both patients (243 µm in case 2 and 238 µm in case 3) [5].

## Discussion

The original description of congenital hamartoma of the RPE was given by Laqua in 1981, based on his clinical observations in two cases [6]. In 1989, Gass published a review on focal congenital anomalies of the RPE [1]. He described RPE hamartomas as focal, nodular jet black lesions that involve the full thickness of the retina and spill onto the inner retinal surface in an umbrella-like fashion. In the largest series – 5 cases – by Shields et al., most cases were asymptomatic due to extrafoveal location [7]. The authors noted subtle feeder vessels clinically in all five patients (which were not seen angiographically), surface wrinkling in four patients, and vitreous cells and exudation in one patient. All patients were stable over 1–16 years of follow-up. Sub-foveal location of the hamartoma results in poor vision [8]. In our first case, the hamartoma clinically mimicked an IOFB; easily differentiated by B-scan ultrasonography and X-ray. Further, EDI-OCT findings in all the cases were highly characteristic, similar to the findings previously reported on time domain OCT [2], [3], [8]. However, differentiation from the more closely mimicking diseases, i.e. CHRPE and CHRRPE, may be tricky. The EDI OCT and FAF findings of presumed congenital simple RPE hamartoma are however different from these lesions. CHRPE shows increased reflectivity of the RPE layer with thinning of overlying retina and loss of photoreceptors in the region of the flat CHRPE and normal underlying choroid on EDI OCT [2], [3]. CHRRPE shows a prominent epiretinal membrane with thickening and disorganization of the underlying retina, and typical saw-tooth or folded patterns of vitreoretinal traction on OCT [2], [3]. Fundus autofluorescence of CHRPE shows marked hypoautofluorescence similar to simple RPE hamartoma in about half of the cases; the lacunae are however common, and reveal mild-moderate hyperautofluorescence attributable to bare sclera [4]. CHRRPE shows hypofluorescence corresponding to heavily pigmented lesion containing melanin, and fuzzy hyperautofluorescence corresponding to the epiretinal membrane [9]. We present the second largest series of presumed congenital simple RPE hamartoma, and – to the best of our knowledge – the first report of FAF findings in this tumor. To summarize, multimodal imaging with FAF and EDI-OCT can help to differentiate simple RPE hamartoma from similar RPE lesions, and may serve as a useful adjunct to clinical diagnosis of this rare tumor.

## Notes

### Competing interests

The authors declare that they have no competing interests.
